# COVID-19 and flu vaccination in Romania, post pandemic lessons in healthcare workers and general population

**DOI:** 10.1371/journal.pone.0299568

**Published:** 2024-03-07

**Authors:** Mădălina Adriana Malița, Loredana Sabina Cornelia Manolescu, Viorel Ștefan Perieanu, Iuliana Babiuc, Elena Cristina Marcov, Camelia Ionescu, Irina Adriana Beuran, Irina Prasacu, Mădălina Violeta Perieanu, Ioana Voinescu, Mihaela Corina Radu, Liliana Burlibasa, Anca Irina Dumitrescu, Mihai Burlibasa

**Affiliations:** 1 Department of Dental Technology, Faculty of Midwifery and Nursing, “Carol Davila” University of Medicine and Pharmacy, Bucharest, Romania; 2 Department of Microbiology, Parasitology and Virology, Faculty of Midwifery and Nursing, “Carol Davila” University of Medicine and Pharmacy, Bucharest, Romania; 3 Department of Restorative Dentistry, Faculty of Dentistry, “Carol Davila” University of Medicine and Pharmacy, Bucharest, Romania; 4 Department of Dental Prostheses, Faculty of Dentistry, “Carol Davila” University of Medicine and Pharmacy, Bucharest, Romania; 5 Department of Fundamental Sciences, Faculty of Pharmacy, “Carol Davila” University of Medicine and Pharmacy, Bucharest, Romania; 6 Department of Genetics, Faculty of Biology, University of Bucharest, Bucharest, Romania; Korea Disease Control and Prevention Agency, REPUBLIC OF KOREA

## Abstract

**Background:**

Influenza and corona viruses generate vaccine preventable diseases and have pandemic potential, frequently dramatic. A co-infection with these viruses, may be a new worldwide threat, researchers name it flurona. The aim of our study is to assess flu and COVID-19 Romanian vaccination for 2022–2023 season and the factor associated with higher odds to receive flu and COVID-19 vaccine.

**Methods:**

An analytical cross-sectional observational survey was conducted in the general population; a self-administered questionnaire was used.

**Results:**

1056 responders were analyzed, mean age 32.08 ±13.36 years (limits:18–76), majority, 880 (83.33%), from urban areas, 608 (57.58%), high school graduated, 400 (37.88%) parents. More than half of the responders were healthcare workers, 582 (55.11%), also considered study population. In the study group, 796 (73.37%) responders consider flurona vaccination useful; and 872 (82.57%) responders consider that no sanctions are needed for not flurona vaccinating. In the 2022–2023 season, 162 (15.34%) responders vaccinated against the flu and 300 (28.41%) against COVID-19. The factor associated with higher odds to receive flu and COVID-19 vaccine was the habit of flu vaccination: for flu (OR = 58.43; 95% CI: (34.95–97.67)) and for COVID-19 (OR = 1.67; 95% CI: (1.21–2.31)). Other factors such as having university degree (OR = 1.46; 95% CI: (1.08–1.98)) and being a healthcare worker, (OR = 1.41; 95% CI: (1.07–1.87)) were influencing factors only for adult COVID-19 vaccination in the 2022–2023 season. In the parents’ group, in 2022–2023 season, only 48 (12%) vaccinated their children against the flu and 68 (17%) against COVID-19, mostly parents that vaccinated themselves, p<0.001. In the 2022–2023 season, there were only 82 (7.65%) responders vaccinated against both diseases. Logistic regression analysis showed that no factor analyzed influenced the flurona vaccinated parent’s decision to vaccinate their children for flu and for COVID-19.

**Conclusions:**

In the season 2022–2023, in Romania, the vaccination against flu and COVOD-19 is low, in adults and children as well. More efforts must be done to increase flurona vaccination, public health educational programs are strongly needed. Children, that are at greater risk when co-infecting with these viruses, must be vaccinated, school vaccination programs should be considered.

## Introduction

Respiratory transmitted viruses, such as coronaviruses and influenza, are viruses with pandemic potential. The fall winter season is perfect for them to thrive. Due to cold weather and clustering of people in doors these viruses spread easily. Two types of respiratory viruses have shown a particular ability in generating overwhelming pandemics: flu type A[H1N1] and SARS CoV-2. A simultaneous viral infection of both these viruses have been named flurona [1, 2]. These viruses develop vaccine preventable diseases namely influenza and COVID-19 [3]. While there is no new vaccine combined for both influenza and COVID, there are separate vaccines for influenza and COVID. A famous influenza pandemic, very well acknowledged, from 1918, is known as the Spanish flu-18 [4]. It has an estimated projection of 40–50 million deaths. The last global influenza pandemic started in April 2009. It ended in August 2010, with a total of 18 500 laboratory-confirmed deaths around the world and an estimated total real toll death in between 105,700 to 395,600 deaths [5]. The responsible virus was influenza A[H1N1] pdm09, a strain with higher mortality in children, young adults, and pregnant women [6]. In November 2002, another type of respiratory virus had emerged into a pandemic. Until July 2003, it caused 774 deaths worldwide by severe acute respiratory syndrome (SARS). The responsible virus was a coronavirus [7]. This virus was a promising pandemic virus, but nobody has expected its successor, SARS CoV-2 virus, a member of the same family. Every year we receive indication of the circulating flu viruses in both hemi-spheres. This comes from GISRS (Global Influenza Surveillance and Response System). GISRS is an organization led by WHO. GISRS is used for international virological and epidemiological surveillance of human influenza [8]. The organization is composed by 150 laboratories in 136 countries. Its purpose is to “decide which strains will be contained into the annual flu vaccines. According to this recommendation, medical doctors start vaccination against the flu. Every year the at-risk population is targeted [3], recommendation toward vaccination is given to the general public. Since 2020 several vaccines were approved and used in many countries across the world against COVID-19 [9–12]. Initially two doses were recommended as the correct algorithm of COVID-19 vaccination. In time, the booster doses became more and more needed [13–15]. Available data from previous studies on vaccine coverage showed differences according to country and period. For example, in a 2023 study from Saudi Arabia, more than 80% of the participants, (out 422 participants), strongly agreed that flu and COVID-19 vaccines must be mandatory for all populations [16]. In an US 2023 study, regarding COVID-19 vaccination for children under the age of five years, from 591 participating parents, 49% indicated that they intended to vaccinate their child(ren) while 29% reported that they would not [17]. A recent consistent study from Europe (7018 adult participants from Poland), showed that COVID-19 vaccinations levels were high. In this study 81.82% (n = 5742) were vaccinated and 18.18% (n = 1276) were not [18]. Factors associated with lower odds to receive the vaccine were: being men (p = 0.02; OR = 0.72–0.97), having lower education status (p = 0.001, OR = 0.56–0.77), living in a smaller residence area (p < 0.001, OR = 0.47–0.73.), not receiving flu vaccination (p < 0.001, OR = 24.51) and not using health monitoring applications (p < 0.001, OR = 1.56) [18]. Regarding flu vaccination, recent US studies from 2023 showed different levels of vaccine hesitancy. For example, 49% (out of 700 participants) respondents were classified as having general vaccine hesitancy, 17% did not receive COVID-19 vaccine, and 36% did not receive flu vaccine [19]. In the beginning of the COVID-19 pandemic, in Italy, people were willing to start receiving flu vaccine. More than 50% (53.8%; n = 2214) of the 4116 studied sample from Italy were willing to receive flu vaccinations contraries to previous seasons. Few people rejected COVID-19 vaccine, only 17.5% of respondents stated that it was unlikely that they would accept a future COVID-19 vaccine (n = 720). Reasons behind vaccine hesitancy were mainly a lack of trust in the vaccine (41.1%), the fear of side effects (23.4%), or a lack of perception of susceptibility to the disease (17.1%). Reasons for vaccinating both against flu and COVID-19 were a higher knowledge score about SARS-CoV-2/COVID-19 and at least one flu vaccination during previous influenza outbreaks [20].

In the end of the COVID-19 pandemic, in Spain, vaccination coverage against SARS-CoV-2 was very high (97.8% out of 1894). This was especially in flu vaccinated students, while flu vaccination coverage was low and increased only from 26.7% to 35.0% (p < 0.05), [21].

Other recent studies showed vaccine conditions in different populations, including general population, university students, healthcare workers, outpatients, and parents [22–29].

In Romania, several recent studies have reported low levels of vaccination, for COVID-19, [9, 11, 12], as well as for flu [30]. In Romania there are not many studies regarding Romania flu vaccination. The fall-winter season of 2022–2023 came with more respiratory viral infections than ever. The reasons may be increased replication of respiratory viruses in cold temperatures and week immunity in general population. Week immunity may be due to mask wearing, officials of WHO declared [3]. Coinfection of flu and coronaviruses is not new, since the beginning of the COVID-19 pandemic, medical community have been preparing for it [31, 32]. Previous recent pandemics, have shown us the importance of having vaccination as a preventing tool [33–35]. According to CDC, (Centre for Disease Control and Prevention), from Atlanta, U.S.A. and ECDC, (European Centre for Disease Prevention and Control, from Stockholm, Sweden the recommendations for the 2022–2023 current season are to vaccinate against both influenza and COVID-19. The indication is stronger for vulnerable population groups (such as healthcare workers, people over 60 years of age, pregnant women, and those with comorbidities and/or underlying conditions) [36, 37]. This comes in the context of a new coronavirus, Omicron XBB1.5 sub-lineage spreading [38]. Since October 2022 an alarm was issued at European level about the risk of severe pressure on healthcare systems due to flu and COVID-19 co-circulation [39]. Taking all of this information into account, the aim of our research is to assess flu and COVID-19 Romanian vaccination for 2022–2023 season and the factor associated with higher odds to receive flu and COVID-19 vaccine, e.i., flurona vaccination.

## Materials and methods

### Study design, setting, and participants

An analytical cross-sectional observational survey was conducted in Romania in the fall-winter period from the 5^th^ of October 2022 to the 27^th^ of January 2023 in general population, (all people living in the Romanian forty-one counties and the capital). The inclusion criteria for the participants were to be at least 18 years of age, and to have Romanian citizenship. For an accurate design, we used “The Strengthening the Reporting of Observational Studies in Epidemiology” (STROBE) recommendations. The STROBE checklist is included in the Supplementary Materials (S1 File). To be sure that we have a representative sample, we stratified sampling to preserve known groups that are important to the study, such as vulnerable people, (old aged). As a sampling procedure, or sample size calculation we applied systematic sampling. We broke down the Romanian population into the 41 counties and the Capital. On the formed groups we used simple random sampling. We selected two settlements in each county, as the area of setting or residence, one town and one village. To cover urban and rural areas we considered urban area the towns, and rural area the villages. Then we used the population evidence from town halls for each county to form the final list for a 25 simple random sample of one county. The service of population evidence provided us with the contacts of the randomly selected individuals. The study was conducted with the aid of medical students’ volunteers from the “Carol Davila” University of Medicine and Pharmacy from Bucharest, Romania. The goal of attendance was at least 1000 participants and at least 25 responders from each county. We did not aim for a certain occupation (activity domain), all areas of employment were welcomed and unemployed people were also admitted. Still, we put the emphasis on one domain of activity of the participants: healthcare workers, (HCW), due to the fact that HCW are a category exposed at risk of getting flu and COVID-19 more easily and, they have the knowledge about these diseases. Also, HCW, who were also considered study population, may have the power to advise the general population toward vaccination. We considered as HCW the following categories: medical doctors, dentists, nurses, pharmacists, paramedics, midwives, kineto-therapists, dental technicians.

The level of education, (the level of studies), was considered as follows when we analyzed the whole group: general education: people with primary and gymnasia education, high school education: people with high school studies and college or secondary school, and superior education: people with faculty, university degree, PhD or even Post Doc.

**Fig 1:**
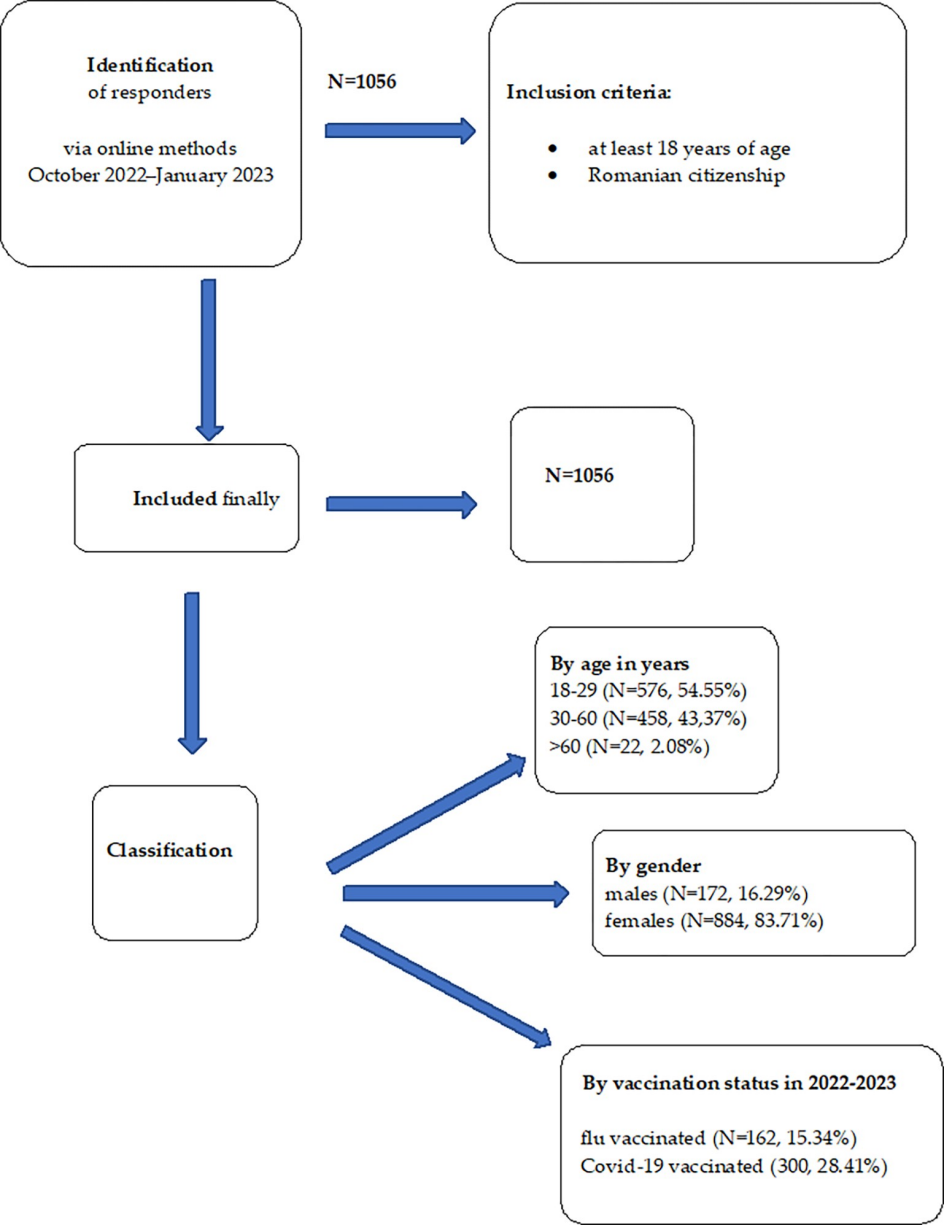
Data selection process.

We did not restrict our target population by administering the questionnaire only to people with children, all people were included irrespective of parental status. Initially 1060 participants responded to the questionnaire, but we had to eliminate four responders because they were not 18 years of age. The full participants’ selections process is presented on the Fig 1.

### Survey questionnaire and data collection

A self-administered questionnaire was used to gather the data. The questionnaire was administered online by medical students that were volunteers and went into the territory. They helped the targeted population to answer the online questionnaire by providing them the questionnaire. We used the Google Forms cloud-based survey software and applied the questionnaire online in: October 2022, November 2022, December 2022 and January 2023. It included 26 multiple-choice questions (S2 File). After it was issued in Google Forms, written informed consent was required and obtained from each responder. All questions were mandatory. Here are the main topics covered in the questionnaire: socio-demographic and educational characteristics, occupation (with special attention toward medico-social staff: doctors, social workers, medical assistants, pharmacists, nurses, dental technicians, midwives, paramedics, physiotherapists), being diagnosed and being vaccinated for flu and COVID-19. In order to achieve clarity, the questionnaire was initially tested as a pilot study. We identified and resolved all problems before implementing the full survey, such as validity of each question, question flow, response rate, average time for completing the questionnaire, instances of confusion and question types. The pilot study of our questionnaire was tested on a sample size representative of the target population and after completing the pilot study the respondents offered their feedback.

### Ethical approval

The questionnaire was peer-validated and approved by the Ethics Committee of the Obstetrics and Gynecology Hospital, Ploiesti, Romania (49739/29.09.2022); all the procedures in the study respected the ethical standards of the Helsinki Declaration. Written informed consent was compulsory.

### Statistical analysis

The data from the questionnaire were analyzed by means of the Microsoft Office package Excel and IBM® SPSS® Statistics Version 23.0 software. For data processing, the COUNTIFS function in Excel was used to filter and sort the initial database. The decision regarding vaccinating (annually, only in pandemic, intention, already vaccinated) in people having children was considered the most important variable and was, hence, considered as the main dependent variable for consecutive analysis. Applied tests were descriptive ones. Since all data, except for age, were categorical variables, we also applied the nonparametric Chi square test. The variables considered significant in relation to our main dependent variable were included in a logistic regression to identify the most important predictors for deciding to vaccinate children. For all tests, the threshold for statistical significance was 0.05.

## Results

### Genal data and vaccination habbits in the group of responders

There were 1056 completed questionnaires. We draw complete evidence of all the answers in the S2 File. The responder’s group profile was as follows: mean age 32.08 ±13.36 years (limits:18–76), majority women, 884 (83.71%), from urban areas, 880 (83.33%) and only 474, (44.89%) responders had other occupations than HCW (activity domain from the medical area). A little more than half of the responders, 608 (57.58%), fit into the category of high school education, there were 44 (4.16%) responders that graduated only primary school. Some responders were parents: 400 (37.88%), in the entire group: 206 (19.51%) responders had one child, while 196 (18.56%) responders had at least or more than two children. 260 (24.62%) responders consider that flurona vaccination (vaccination against the flu and COVID-19) is useless, while 872 (82.57%) consider that no sanctions must be introduced in the vaccination law if people refuse flurona vaccination. The general characteristics of our responders are presented in Table 1. For establishing that a responder had flu or COVID-19 and recovered he or she must have a medical record of a positive laboratory RT-PCR test. In our 1056 initial group, the reasons declared by the responders for not vaccinating against flurona were adverse events due to vaccine administration 286 (27.08%), lack of proper information about the benefits of specific flu and COVID-19 vaccination 286 (27.08%) and costs 8 (0.75%).

**Table 1:** General socio-demographic and educational characteristics of respondents to the questionnaire. HCW = healthcare workers.

Characteristic	N = 1056 (100%) All studied responders	N = 582 (55.11%) HCW	N = 474 (44.89%) General population
age in years, median (min-max)	26 (18–76)	28(18–62)	23(18–76)
masculine gender	172 (16.29%)	110(18.9%)	62(13.08%)
rural residence	176 (16.67%)	92(15.81%)	84(17.72%)
not parents/without children	656 (62.12%)	354(60.82%)	302(63.71%)
university graduated	404 (38.26%)	226(38.83%)	178(37.55%)
had flu and recovered	162 (15.34%)	90(15.46%)	72(15.19%)
had COVID-19 and recovered	432 (40.91%)	234(40.21%)	198(41.77%)
consider flurona vaccination useful	796 (73.37%)	452(77.66%)	344(72.57%)
support introduction of sanction for not flurona vaccinating	184 (17.42%)	114(19.59%)	70(14.77%)

We evaluated our 1056 responders study group vaccination habits toward flu and COVID-19 vaccination, for the responder’s personal outcome Table 2. We considered the fact that in the 1056 responders’ group there were 582 HCW.

**Table 2:** The total 1056 responders’ group, (582 HCW), practice toward flurona vaccination.

attitude	for flu vaccination 1056 group (%)	for flu vaccination 582 HCW (%)	for COVID-19 vaccination 1056 group (%)	for COVID-19 vaccination 582 HCW (%)
vaccinate each year	226 (21.40%)	158 (27.14%)	48 (4.55%)	28 (4.81%)
vaccinate only in pandemic	272 (25.75%)	148 (25.42%)	762 (72.16%)	438 (75.25%)
do not vaccinate at all	558 (52.84%)	276 (47.42%)	246 (23.30%)	116 (19.93%)
vaccinated last season (2021–2022)	264 (25.00%)	176 (30.24%)	864 (81.82%)	508 (87.28%)
vaccinated this season (2022–2023)	162 (15.34%)	112 (19.24%)	300 (28.41%)	186 (31.95%)
with intention to vaccinate this season (2022–2023)	256 (24.24%)	146 (25.08%)	106 (10.04%)	70 (12.02%)

### Situation of flu and COVID-19 vaccination in HCW

In the 582 HCW group of responders, only 158 (27.14%) HCW vaccinated each year for flu, and 276 (47.42%) HCW did not vaccinate at all. The vaccination trend against flu in the HCW responders’ group was low, with 176 HCW flu vaccinated last season and only 112 HCW flu vaccinated this season. Regarding COVID-19 vaccination we noticed that the majority of HCW responders prefer to vaccinate in the pandemic, 438 (75.25%) HCW responders, and only 186 (31.95%) HCW responders COVID-19 vaccinated this season comparing with 508 (87.28%) HCW, that vaccinated last season.

In both seasons, after applying the nonparametric Chi square test for the 1056 group, we found a strong association between being vaccinated and being an HCW, for flu vaccination (p = 0.001) as well as for COVID -19 vaccination (p = 0.001).

### Situation of flu and COVID-19 vaccination in the last season, 2021–2022

In 2021–2022 season, the COVID-19 pandemic period, for the 1056 studied group there was no association between the flu vaccination and age, area of residence (p = 0.252), gender (p = 0.181) or education (p = 0.201). During this period, the last season, 2021–2022, the level of studies influenced COVID-19 vaccination, p = 0.001. The area of residence, (urban area or rural area), was a factor of COVID-19 vaccination, more people from the urban area vaccinated against COVID-19 last season than the people from rural areas, (p = 0.032). We found no association between age, gender (p = 0.870) and COVID-19 vaccination for the 2021–2022 season.

### Situation of flu and COVID-19 vaccination in the current season, 2022–2023

In the 2022–2023 season, 162 (15.34%) adults responders vaccinated against the flu and 300 (28.41%) against COVID-19. We were surprised to see that despite the flurona threat, in the present 2022–2023 season, there were not many flurona vaccinated people. In the 2022–2023 season, compared with the 2021–2022 season, the level of studies did not influence COVID-19 vaccination, p = 0.240.

We run the nonparametric Chi square test to study if being diagnosed with influenza or COVID-19 influenced the adult’s vaccination or the intention to vaccinate this season, 2022–2023. We discovered that there is a strong connection between a previous diagnose of influenza, p = 0.006; or a previous diagnose of COVID-19, p = 0.025; and the intention to vaccinate for this season.

Logistic regression applied on the 1056 responders’ group revealed after stepwise selection that the most important factor as adults flu vaccinating influencing factor was habit of flu vaccination (annual vs. only in pandemic) (OR = 58.43; 95% CI: (34.95–97.67)). We calculated the odds ratio (OR) and confidence interval at the 95 Level (CI), Table 3, for the actual 2022–2023 season. Other factors that were not as relevant are living in the urban area (OR = 1.66; 95% CI: (0.89–3.10)) and working in the medical domain (OR = 1.23; 95% CI: (0.76–1.99)). For adult COVID-19 vaccination, Table 4, the factors that influenced vaccination on the 2022–2023 season were: the habit of flu vaccination, (OR = 1.67; 95% CI: (1.21–2.31)), having university degree (OR = 1.46; 95% CI: (1.08–1.98)) and being a HCW, (OR = 1.41; 95% CI: (1.07–1.87)).

**Table 3:** Logistic regression for the 1056 total studied group for flu vaccination in the 2022–2023 season.

	*P*	OR	Lower CI 95%	Upper CI 95%
Activity domain (HCW vs. Not HCW)	0.387	1.23	0.76	1.99
Age (lower age)	0.331	0.78	0.48	1.27
Geographic area (urban vs. rural)	0.107	1.66	0.89	3.10
Education (superior education vs. high school education)	0.603	0.87	0.53	1.44
Diagnosed with flu in the past	0.056	0.56	0.31	1.01
Habit of flu vaccination (annual vs. only in pandemic)	< 0.001	58.43	34.95	97.67

Legend: *P* (*P* -value), OR (odds ratio), CI (confidence interval)

**Table 4:** Logistic regression for the 1056 total studied group for COVID-19 vaccination in the season 2022–2023.

	*P*	OR	Lower CI 95%	Upper CI 95%
Activity domain (HCW vs. Not HCW)	0.014	1.41	1.07	1.87
age	0.001	0.60	0.45	0.82
Geographic area (urban vs. rural)	0.331	1.20	0.82	1.76
Education (superior education vs. high school education)	0.014	1.46	1.08	1.98
Diagnosed with flu in the past	0.918	0.98	0.67	1.42
Habit of flu vaccination (annual vs. only in pandemic)	0.002	1.67	1.21	2.31

Legend: *P* (*P* -value), OR (odds ratio), CI (confidence interval); HCW = health care workers

### Parents attitude regarding their children vaccination

In our studied of 1056 responders’ group, there were 400 parents. We evaluated the parent’s attitude regarding their children vaccination against flu and COVID-19. We found after applying the nonparametric Chi square test no association of the children vaccination with the parent’s medical practice, being a healthcare worker in the medical domain did not influence the children vaccination, (p = 0.923).

In the 582 HCW group only 228 had children. We analyzed the way in which healthcare workers regarded their children vaccination, Table 5. We discovered that only a few HCW responders vaccinated their children, 36 healthcare workers vaccinated their children for flu and 38 healthcare workers vaccinated their children for COVID-19, and the majority of HCW had no intention to vaccinate their children: 182 (79.82%) HCW that were parents had no intention to vaccinate their children against flu and 198 (86.84%) HCW responders that were parents had no intention to vaccinate their children against COVID-19. But when comparing all 400 parents with the nonparametric Chi square test, we saw that the 228 HCW parents have vaccinated statistically significative more children than the other parents against the flu, 36 children vs 12 children, p = 0.022; comparing with vaccination against COVID-19, 38 children vs 30 children, p = 0.653, COVID-19 vaccination was similar for all parents. Nevertheless, children vaccination rates are small.

**Table 5:** The parent’s group, total 400 responders, (228 HCW), attitude toward flurona vaccination.

attitude	for flu vaccination 400 total parents (%)	For flu vaccination 228 HCW parents (%)	For COVID-19 vaccination 400 total parents (%)	For COVID-19 vaccination 228 HCW parents (%)
vaccinate their child/children each year	160 (40%)	70 (30.70%)	32(8%)	6 (2.63%)
vaccinate their child/children only in pandemic	100 (25%)	24 (10.53%)	270(67.5%)	124 (58.77%)
do not vaccinate at all their child/children	140 (35%)	134 (58.77%)	98(24.5%)	98 (42.98%)
vaccinated their child/children this season (2022–2023)	48 (12%)	36 (15.79%)	68(17%)	38 (10.67%)
with intention to vaccinate their child/children this season (2022–2023)	78 (19.5%)	46 (20.17%)	46(11.5%)	30 (13.16%)

In the parent’s group in the 2022–2023 season, there were no correlation between the children vaccination and the level of education, neither for influenza, 48 parents (18 parents with high school education vs 30 parents with superior education, p = 0.453) or for COVID-19, 68 parents (34 parents with high school education vs 34 parents with superior education, p = 0.181). So, the level of studies of the parents did not influence them in vaccinating their children. The area of residence, (the place where they live), did not influence children’s vaccination, either for flu, 48 parents (12 parents from rural vs 36 parents from urban area, p = 0.592), or for COVID-19, 68 parents (from12 rural vs 56 parents from urban area, p = 0.923).

But we found that the parents that vaccinated themselves (N = 74 for flu and 104 for COVID-19), were more willingly to vaccinate their children this season, 2022–2023 for both infectious diseases discussed: for influenza, N = 48 parents, p = 0.001 and for COVID-19, N = 68 parents, p = 0.001.

### Flurona vaccinated group, vaccinated against flu and COVID-19 at the same time

From the total 1056 responders there were 247 (23.39%) flurona vaccinated (vaccinated against flu and COVID-19 at the same time) in the 2021–2022 season and 82 (7.65%) responders were flurona vaccinated in the current, 2022–2023 season. The general socio-demographic, educational and vaccination characteristics are presented in Table 6.

**Table 6:** General socio-demographic, educational and vaccination characteristics of flurona respondents to the questionnaire.

Characteristic	2021–2022 flurona vaccinated N = 247(%)	2022–2023 flurona vaccinated N = 82(%)
age, in years, median (min-max)	32 (18–76)	25 (18–67)
masculine gender	50 (20%)	14 (17.07%)
rural residence	46 (18.4%)	12 (14.6%)
healthcare workers	170 (68%)	60 (73.17%)
not parents/without children	128 (51.2%)	52 (63.41%)
university graduated	100 (40%)	36 (43.9%)
had flu and recovered	60 (24%)	18 (21.95%)
had COVID-19 and recovered	136 (54.4%)	38 (46.34%)
consider flurona vaccination useful	236 (94.4%)	80 (97.56%)
support introduction of sanction for not flurona vaccinating	92 (36.8%)	46 (56.09%)
flu vaccinate each year	184 (73.6%)	68 (82.93%)
flu vaccinate only in pandemic	48 (19.2%)	14 (17.07%)
flu vaccinate their child/children each year	106 (42.4%)	38(46.34%)
flu vaccinate their child/children only in pandemic	28 (11.2%)	4 (4.88%)
do not flu vaccinate at all their child/children	36 (14.4%)	6 (7.32%)
do not COVID-19 vaccinate at all their child/children	52 (20.8%)	14 (17.07%)
flu vaccinated their child/children this season (2022–2023)	38 (15.2%)	18 (21.95%)
COVID-19 vaccinated their child/children this season	26 (10.4%)	18 (21.95%)
with intention to flu vaccinate their child/children	52 (20.8%)	12 (14.63%)
with intention to COVID-19 vaccinate their child/children	28 (11.2%)	8 (9.76%)

We tried to find out which were the factors that influenced the children vaccination for this season, 2022–2023, in the 82 responders group that flurona vaccinated 82 responders group presented ahead in Table 6. We applied logistic regression, Table 7. We analyzed the children’s vaccination against the flu and COVID-19 for this season.

**Table 7:** Logistic regression model of the flurona vaccinated adults that vaccinated their children against the flu in the 2022–2023 season.

	*P*	OR	Lower CI 95%	Upper CI 95%
Activity domain (HCW vs. Not HCW)	0.684	0.65	0.08	5.07
Education (superior education vs. high school education)	0.335	0.37	0.05	2.77
Habit of flu vaccination (annual vs. only in pandemic)	0.282	0.25	0.02	3.05

Legend: *P* (*P* -value), OR (odds ratio), CI (confidence interval); HCW = health care workers

In the 82 flurona vaccinated responders, logistic regression analysis showed that no factor analyzed influenced the flurona vaccinated parent’s decision to vaccinate their children for flu and for COVID-19, Table 7.

## Discussion

Flu and COVID-19 are very present worldwide, and doctors named this simultaneous threat flurona [40]. There is no mentioning on WHO, CDC or ECDC sites about it, but since 2020^th^ the medical community have wondered, researched, and discussed all cases of simultaneous flu and SARS CoV-2 infection, namely flurona. In the early 2020s there were few cases of flurona [41, 42], but later, as COVID-19 pandemic progressed, more and more cases of flurona appeared [43], generating for the 2022–2023 current season an official indication of vaccination against both diseases [3, 36, 37]. Systematic literature reviews [43], described 79 flurona patients in eleven prevalence studies published in Medline, Web of Science, and Embase databases from December 2019 to September 2020. Until July 2021, twelve literature review studies from PubMed, Web of Science, Embase, Cochrane Library and China National Knowledge Infrastructure Database (CNKI) revealed another 9,498 flurona patients [44].

Flurona (coinfection of SARS-CoV-2 and influenza), in this fall-winter season is no doubt a major concern in the medical community, with significant impact on morbidity, mortality and health-service request. Studies from 2021 have shown that the patients with a flurona coinfection had a risk of death of 5.92 (95% C.I.: 3.21–10.91) times higher than those without influenza or SARS-CoV-2 alone [45]. In U.S. studies from early 2022, have shown that flurona cases reported higher rates of symptoms, (including congestion, cough, fever/chills, headache, myalgia/arthralgia, pharyngitis, and rhinitis [46]), but were not life threatening in relatively young, healthy patients. CDC surveillance platforms reported during the 2021–2022 season that flurona infection occurred in young patients who had been hospitalized or died. Flurona coinfection occurred in 6% (32 of 575) of pediatric hospitalizations and in 16% (seven of 44) of pediatric deaths [47]. In this report among the seven coinfected patients who died, none was flurona vaccinated.

Vaccination, both against influenza and COVID-19, remains a powerful weapon in the flurona prophylaxis. Worldwide in the season 2022–2023 vaccination remain the most important and reliable method of preventing flurona infections. All other preventive measures, such as wearing the mask or social distancing, have decreased their powers [3].

In Romania, the National Public Health Institute reports each week the level of respiratory infectious diseases in the general population. For the second week in January 2023 there were 139.255 cases of respiratory infections, with 35.4% more than the former week. Out of these cases, 5.082 were flu clinical cases and 68 severe respiratory infections (SARS); 381 cases were laboratory diagnosis confirmed as follows: 46 influenza type A subtype H3, 102 influenza type A subtype H1 and 226 influenza type A without subtyping, 6 type B without subtyping and one coinfection type A and B [48] The number of flurona cases was double from the former week. At the national level, until the 15th of January 2023, for the flu season 2022–2023, the National Health Institute reported 1,154 people with laboratory-confirmed influenza, 26 flurona cases and a total of 27 associated deaths [48]. Four deaths were attributed to flurona and were reported in young people under 18 years.

Since the beginning of the season until the 15^th^ of January 2023, 1,446,572 people have been flu vaccinated in Romania with vaccine distributed by the Ministry of Health. These people were from the risk groups. Currently, according to worldometer, Romania has a population number of 18,916,885, [49].

Our study aimed to draw a picture of the general situation in Romania regarding flu and COVID-19 vaccination and discover the factors that may influence flurona adult and children vaccination in the 2022–2023 season. We analyzed 1056 Romanian people from different populational categories, with various ages and areas of residence. We studied their profile, their flurona vaccination habits and their children vaccination outcomes on the background of increasing cases of flurona in Romanian children and potentially deadly outcome.

Overall, 1056 studied group, only 82 (7.65%) responders were flurona vaccinated in the current, 2022–2023 season. This is a very low percent compared with previous 2021–2022 Romanian COVID-19 vaccination studies, [9, 11]. These responders average age was 32.34 ±14.45 years, (limits 18–67), majority women, 68 (82.93%), from urban areas 70 (85.36%), 60 healthcare workers (73.17%) and 52 (63.41%) did not have children. In these 82 flurona vaccinated responders, only 18 (21.95%) had flu and recovered and 38 (46.34%) COVID-19. Despite the fact that they were all flurona vaccinated and the majority, (80, 97.56%), considered flurona vaccination useful and supported introduction of sanctions for not flurona vaccinating (46, 56.09%), only 18 (21.95%) had flurona vaccinated their child(ren) this season (2022–2023). Even fewer, 12 (14.63%), had intention to flurona vaccinate their children. The majority, 68 (82.93%), declared that in the past they had the habit to flu vaccinate each year. There are few studies on flu vaccination and general vaccination in Romania.

Worldwide, in the current season, 2022–2023, the opinions, attitudes and willingness of people toward influenza and COVID-19 vaccination have changed. A recent review consisting of eighty studies and published this year reviled the patient-reported attitudes towards influenza vaccination in the adult general population from 16 countries. Negative attitude towards healthcare workers and medical domain was the first barrier to vaccinate (31.1%), while the first facilitator of influenza vaccination was trust in HCW (62.0%) [50]. Our study of the group of 1056 Romanian people revealed by logistic regression analysis that being a HCW positively influence only COVID-19 adult vaccination (OR = 1.41; 95% CI: (1.07–1.87)) and did not influence the flurona children vaccination or adult flu vaccination at all.

Different theories (e.g., theory of planned behavior, protection motivation theory) have been used to examine the vaccine willingness. This can help future studies to take action when they want to adopt a theory to reexamine the present study’s findings [51–55].

Another factor that may influence vaccination against influenza was being previously diagnosed with COVID-19, for example in a study from Greece that took place in September 2022, out of 861 nurses, 57.3% were willing to accept the influenza vaccine, 19% were hesitant, and 23.7% were unwilling, older age being the first positive factor toward vaccination [56]. These findings are not similar with our study.

For the 2022–2023 season a recent study from China, (17,832 healthcare workers), showed by multivariate logistic regression analysis that 74.89% participants were willing to receive influenza vaccine and 82.58% of them were willing to recommend [57]. This is far from our results, where in the 582 HCW group of responders, only 158 (27.14%) HCW vaccinated each year for flu, and 276 (47.42%) HCW did not vaccinate at all. For the current season only 112 HCW flu vaccinated themselves and only 36 HCW vaccinated their children while the majority of HCW from our group, 182 (79.82%) had no intention to vaccinate them. COVID-19 vaccination levels had dramatically dropped in HCW, in 2022–2023 season, there were only 186 (31.95%) HCW that COVID-19 vaccinated compared with 719 (70.42%) from a previous Romanian study of HCW from 2021[9].

The habit of annual flu vaccination turned out to be useful in Romanian studied responders. Our study revealed that this habit was a positive factor for influencing adults flu vaccination (OR = 58.43; 95% CI: (34.95–97.67)) as well as COVID-19 vaccination in the current 2022–2023 season, when COVID-19 vaccinations level had dramatically dropped in Romania [48], (OR = 1.67; 95% CI: (1.21–2.31)). However, because influenza viruses undergo rapid and sustained antigenic drift, resulting mismatches may appear between the vaccine strains and the epidemic strain, so a new universal reformulated flu vaccine would be useful in the fight with this virus [58].

COVID-19 is an infectious disease with many implications, it generated the world greatest pandemic ever seen. SARS-CoV-2 has mutated in a short time into genetically distinct variants that were able to escape our immune capacity of innate and adaptive immunity. RNA viruses have the power to mutate more than other viruses, their adaptability was well documented in the context of other pandemics with RNA viruses such as the human immunodeficiency virus (HIV) [33–35] and influenza viruses. As RNA viruses obscure themselves from host immunity, they uncover weak points in their own armory that can inform more comprehensive, long-lasting, and potentially cross-protective vaccine coverage [59].

COVID-19 vaccination generated many debates regarding mRNA vaccines and their adverse effects. A recent study from Saudi Arabia showed in a group of 426 participants, that after three doses of mRNA COVID-19 vaccines 60.6% experienced injection site pain, 43.9% experienced fatigue, headache, and pain, 32.4% muscle and joint pain, and 24.2% experienced increased body temperature and shivering [60].

Healthcare workers from various domains, neonatology, oncology, and especially the ones from the dentistry, are at great risk for flu and COVID-19 exposure, as they may be a source of flurona spread, as studies had revealed [61–64]. Vaccination may have side effects [65, 66] that cause further burden for people, especially healthcare workers. However, even with such side effects, positive effects on quality of life have been found for people who got vaccinated [67–69], the importance of vaccination being proven.

### Limitations of the Study

This study was subject to several potential limitations. The study was limited by its cross-sectional design. The representativeness of the study sample is limited due to the default selection of respondents who have digital skills, as the studied sample mainly analyzed people who could use the internet and electronic documents. Another limitation of this study is that it analyzed only a short period of time of 4 months and included a limited number of participants with children (400, 37.87%). It is possible that people’s acceptability of child vaccination may change over time as further vaccination campaigns may be instituted; therefore, longitudinal monitoring is indicated.

## Conclusions

In the current season, 2022–2023 in Romania, in children and adults, vaccination against flu and COVID-19 is low. More efforts must be made to increase flu and COVID-19 vaccination levels. Public health educational programs are strongly needed. Children, that are at greater risk when co-infecting with these viruses, must be vaccinated, school vaccination programs should be considered.

## Supporting information

S1 File The STROBE checklist. (PDF)

S2 File The administered questionnaire. (PDF)

S3 File The questionnaire responses. (PDF)
