# Supplementary material for: COVID-19 and flu vaccination in Romania, post pandemic lessons in healthcare workers and general population
Source: PLoS One. 2024 Mar 7;19(3):e0299568. doi: 10.1371/journal.pone.0299568 (PMC10919663; doi:10.1371/journal.pone.0299568)
Supplement: S2 File — (PDF) [file pone.0299568.s002.pdf]

Do I get vaccinated against flu and/or against COVID-19?//Anti-influenza vs anti-Covid-19 vaccination in autumn and winter 2022-2023

STUDY ON ANTI-FLU VACCINATION VS. ANTI-COVID-19

INFORMED CONSENT

QUESTIONNAIRE ADDRESSED TO PERSONS OVER 18 YEARS OLD

Hello!

We invite you to contribute to our research on the flu and COVID-19 vaccination in the 2022-2023 season: fears, beliefs, realities. The questionnaire to be completed has several sections and is addressed both to medico-social staff from specialized units: doctors, social workers, medical assistants, pharmacists, nurses, dental technicians, midwives, paramedics, physiotherapists, as well as to the general population. The completion time is approximately 10 minutes. Participation is voluntary and your answers will be treated confidentially, the information you provide will only be used for research purposes. You have the freedom at any time to withdraw from the study, to interrupt or to resume the questionnaire. The research complies with the international ethical recommendations regarding the absolute confidentiality of the data collected in the study as well as the anonymity and safety of the respondents' data. The requirements of EU Regulation 2016/79, regarding the protection of natural persons regarding the processing of personal data and the free movement of such data and of Law no. 506/2004, regarding the processing of personal data and the protection of private life in the electronic communications sector. It is important that during the study you answer the questions as honestly as possible in order to draw correct conclusions.

For any clarification related to this study, during or after its completion, you can contact us by email at loredana.manolescu@umfcd.ro

QUESTIONS

1. Are you part of the medico-social staff: doctors, social workers, medical assistants, pharmacists, nurses, dental technicians, midwives, paramedics, physiotherapists? Yes/no
2. Do you have children: yes/no
3. How many children do you have: ____
4. How old are you? ___
5. Where do you live: rural/urban
6. Level of education: general education/ high school education/ superior education
7. Gender: ______
8. Have you ever been diagnosed with the flu, through laboratory diagnosis? Yes/no
9. Have you ever been diagnosed with COVID-19, through laboratory diagnosis? Yes/no
10. When do you get vaccinated against the flu? Every year/in epidemic, pandemic/I don't vaccinate
11. When do you get vaccinated against COVID-19? Every year/in epidemic, pandemic/I don't vaccinate

12. Did you get vaccinated against the flu last season, 2021-2022? Yes/no
13. Did you get vaccinated against COVID-19 last season, 2021-2022? Yes/no
14. Did you get vaccinated against the flu this season, 2022-2023? Yes/no
15. Did you get vaccinated against COVID-19 this season, 2022-2023? Yes/no
16. When do you vaccinate your child/children against the flu? Every year/in epidemic, pandemic/I don’t vaccinate
17. When do you vaccinate your child/children against COVID-19? Every year/in epidemic, pandemic/I don’t vaccinate
18. Have you vaccinated your child/children against the flu this season? Yes/no
19. Have you vaccinated your child/children against COVID-19 this season? Yes/no
20. If you haven't been vaccinated against the flu, do you intend to get vaccinated this season? Yes/no
21. If you haven't been vaccinated against COVID-19, do you intend to get vaccinated this season? Yes/no
22. If you haven't vaccinated your child/children against the flu, do you intend to get them vaccinated this season? Yes/no
23. If you haven't vaccinated your child/children against COVID-19, do you intend to get them vaccinated this season? Yes/no
24. What is the reason why you did not vaccinate your child/children?___________
25. What do you think about the two vaccines, anti-influenza and anti-COVID-19?___________
26. Do you think there should be sanctions (Vaccination Law) for people who refuse to vaccinate themselves or their children against the flu or against COVID-19? Yes/no
