# Supplementary material for: COVID-19 and flu vaccination in Romania, post pandemic lessons in healthcare workers and general population
Source: PLoS One. 2024 Mar 7;19(3):e0299568. doi: 10.1371/journal.pone.0299568 (PMC10919663; doi:10.1371/journal.pone.0299568)
Supplement: S3 File — (PDF) [file pone.0299568.s003.pdf]

Responses to the questionnaire TOTAL RESPONDERS: 1056

1. Are you part of the medico-social staff: doctors, social workers, medical assistants, pharmacists, nurses, dental technicians, midwives, paramedics, physiotherapists? Yes/no; 582 (55.11%)/474 (44.89)

2. Do you have children: yes/no; 400 (37.88%)/ 656 (62.12%)

3. How many children do you have:

**Number of children**		**P%**
0	654	61.93
1	206	19.51
2	158	14.96
3	30	2.84
4	4	0.38
5	2	0.19
8	2	0.19
total	1056	

4. How old are you?; 32.08 ±13.36 years (limits:18–76)

5. Where do you live: rural/urban; 176 (16.67%)/880 (83.33%)

6. Level of education: general education/ high school education/ superior education; 44 (4.17%)/608 (57.58%)/404 (38.26%)

7. Gender: 884 (83.71%)/172 (16.29%)

8. Have you ever been diagnosed with the flu, through laboratory diagnosis? Yes/no; 162 (15.34%)/894 (84.66%)

9. Have you ever been diagnosed with COVID-19, through laboratory diagnosis? Yes/no; 432 (40.91%)/ 624 (59.09%)

10. When do you get vaccinated against the flu? Every year/in epidemic, pandemic/I don't vaccinate; 226 (21.40%)/272 (25.75%)/558 (52.84%)

11. When do you get vaccinated against COVID-19? Every year/in epidemic, pandemic/I don't vaccinate; 48 (4.55%)/762 (72.16%)/246 (23.30%)

12. Did you get vaccinated against the flu last season, 2021-2022? Yes/no; 264 (25.00%)/792 (75.00%)

13. Did you get vaccinated against COVID-19 last season, 2021-2022? Yes/no; 864 (81.82%)/192 (18.18%)

14. Did you get vaccinated against the flu this season, 2022-2023? Yes/no; 162 (15.34%)/894 (84.66%)

15. Did you get vaccinated against COVID-19 this season, 2022-2023? Yes/no; 300 (28.41%)/756 (71.59%)

16. When do you vaccinate your child/children against the flu? Every year/in epidemic, pandemic/I don’t vaccinate; 160 (40%)/100 (25%)/140 (35%)

17. When do you vaccinate your child/children against COVID-19? Every year/in epidemic, pandemic/I don’t vaccinate; 32(8%)/270(67.5%)/98(24.5%)

18. Have you vaccinated your child/children against the flu this season? Yes/no; 48 (4.55)/ 352 (95.45%)

19. Have you vaccinated your child/children against COVID-19 this season? Yes/no; 68(17%)/332 (83%)

20. If you haven't been vaccinated against the flu, do you intend to get vaccinated this season? Yes; 256 (24.24%)

21. If you haven't been vaccinated against COVID-19, do you intend to get vaccinated this season? Yes; 106 (10.04%)

22. If you haven't vaccinated your child/children against the flu, do you intend to get them vaccinated this season? Yes/no; 78 (19.5%)/322 (80.5%)

23. If you haven't vaccinated your child/children against COVID-19, do you intend to get them vaccinated this season? Yes/no; 46(11.5%)/354(88.5%)

24. What is the reason why you did not vaccinate your child/children?

adverse events due to vaccine administer 286 (27.08%)

lack of proper information 286 (27.08%)

price 8 (0.75%)

25. What do you think about the two vaccines, anti-influenza and anti-COVID-19?

They are useless, 260 (24.62%)

They are useful 796 (75.38%)

26. Do you think there should be sanctions (Vaccination Law) for people who refuse to vaccinate themselves or their children against the flu or against COVID-19? Yes/no;184 (17.42%)/872 (82.57%)
